# Dural Arteriovenous Fistula Presenting as a Rapidly Progressive Thalamic Dementia: A Case Report

**DOI:** 10.7759/cureus.29392

**Published:** 2022-09-21

**Authors:** Abrar Khan, Ahmed Elkady, Mohamed Rahametallah, Majid F Bakheet

**Affiliations:** 1 Department of Neurology, King Abdullah Medical City, Mecca, SAU; 2 Department of Neurology, Saudi German Hospital, Jeddah, SAU

**Keywords:** bilatera thalamic infarct, endovascular interventions, cerebral venous sinus thrombosis (cvst), intracranial dural arteriovenous fistula, rapid dementia

## Abstract

Rapidly progressive dementia is an uncommon neurological presentation and usually needs extensive workup, especially for reversible causes. Dural arteriovenous fistula (DAVF) has been rarely reported as a cause of thalamic dementia, in which bilateral thalamic venous congestion and edema cause dementia that usually progresses rapidly. We present a case of a 45 years-old male who presented with rapidly progressive severe attention and memory impairment over one week. Initial work-up showed bilateral thalamic recent venous infarctions and edema. Extensive work-up revealed an intracranial DAVF with internal deep venous thrombosis. Management with endovascular treatment of DAVF followed by anticoagulation for venous thrombosis leads to improvement of the patient’s clinical condition, particularly memory and attention. In conclusion, DAFV could present with thalamic rapidly progressive dementia due to bithalamic infarctions and edema. Early diagnosis and treatment will reverse the cause and improve the patient's general and cognitive conditions.

## Introduction

A Dural arteriovenous fistula (DAVF) denotes an abnormal direct connection between an artery and a Dural venous sinus, and it accounts for about 10% of cranial vascular malformations [1]. Spectrums of clinical presentations are pervasive, ranging from pulsatile tinnitus to intracranial hemorrhage (ICH). Rapidly progressive dementia could be the primary presentation of DAVF in extremely rare cases [2]. Thalamic dementia, which is a rapidly progressive dementia, could be the clinical consequence of pathology of both thalami, usually secondary to an infarction, especially if bilateral paramedian thalamic nuclei are affected [3]. We report a rare case of rapidly progressive dementia associated with bilateral thalamic venous infarctions due to an intracranial DAVF.

## Case presentation

**Figure 1:**
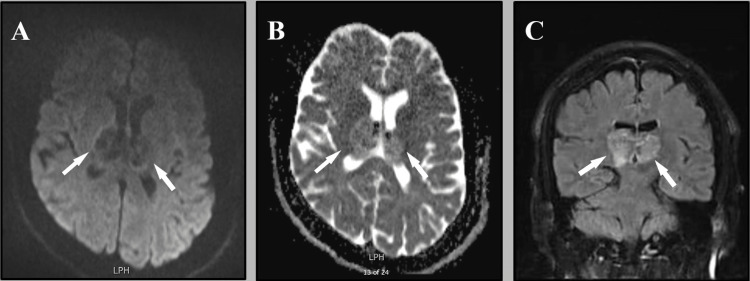
Axial MRI reveals bilateral thalamic edema, which appears as dark in DWI (white arrows) (A), and white in ADC (white arrows) (B). Coronal MRI FLAIR showing bithalamic hyperintense lesion (white arrows) (C).

A 45 years-old man is known to have hypertension, diabetes mellitus, and ischemic heart diseases for years. He presented to our emergency department with worsening attention and memory deficit over one week. The patient was sleepy yet arousable and disoriented to place and time, with both recent and working memory mainly affected. His neurological examination, including cranial nerves, motor power, sensory, and coordination, was unremarkable. His cognitive assessment by The Montreal Cognitive Assessment (MoCA) revealed profound short- and long-term memory impairment with a score of 3 out of 30. Initial laboratory investigations, including CBC and full metabolic profile were within normal, except glycosylated hemoglobin which was 8%. A brain magnetic resonance imaging (MRI) showed bilateral thalamic venous infarctions and edema. (Figure 1).

**Figure 2:**
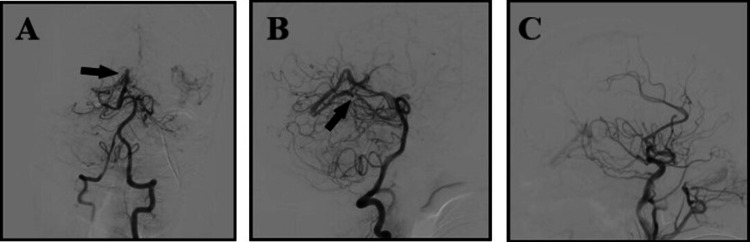
Cerebral angiography showing arteriovenous connection between dural arteries and deep venous system in anteroposterior (A), and lateral (B) views (black arrows), while this shunt disappeared after endovascular intervention (C).

Followed by CT angiography and CT venogram, which showed absent Straight sinus with, raising concern for a DAVF. The following digital subtraction angiography (DSA) confirmed DAVF between straight sinus and Dural Arteries (Figure 2).

Endovascular embolization was performed from the superficial temporal artery of the left external carotid artery without complication. Accordingly, the diagnosis of bithalamic venous infarctions secondary to increased deep venous pressure around the basal ganglia and consequently difficulty of venous drainage due to arteriovenous fistula was done. After securing the DAVF, the patient was started on a full-dose anticoagulant for cerebral venous thrombosis. The patient was discharged from the hospital in a stable condition with mild improvement in his cognitive condition. Follow-up with the patient in an outpatient clinic with MoCA score after two weeks revealed a score of 26 out of 30.

## Discussion

Bilateral thalamic abnormalities can be caused by a wide range of etiologies such as deep venous sinus thrombosis, malignancy, infection, and toxins, thus making a thorough history is essential [4]. Among the vascular etiologies, bilateral thalamic lesions that present with dementia-like symptoms and are caused by DAVFs are rare, while the most common symptom is pulsatile tinnitus [5]. Furthermore, other neurologic deficits, according to the various locations of the DAVF, such as seizure, parkinsonism, and cerebellar symptoms, were reported [6]. Other atypical symptoms, like progressive dementia caused by venous congestion in bilateral thalamic areas, are associated with DAVF, as in our case, so-called thalamic dementia. Thalamic dementia from bilateral paramedian thalamic pathology may be due to the association of the neurological and behavioral disorders related to either left or right paramedian thalamic lesion; however, it has not yet been entirely clarified [4]. Patients who present with progressive dementia should be evaluated carefully for reversible causes, as symptoms could recover after appropriate therapy, like in our patient when an intracranial DAVF was identified and managed by intervention.

Suspicion for an arteriovenous abnormality should be raised if ischemic infarction or ICH is seen in an unusual location or extreme age groups [2]. A high level of doubt should be maintained in the proper clinical context despite the absence of abnormal vessels on noninvasive imaging, as the diagnostic accuracy of noninvasive imaging for DAVF within deep cerebral veins is usually low [7]. DSA remains the gold standard to diagnose and fully define the cerebral lesion and to stratify the risk of rebleeding [3]. Treatment of DAVF should be based on patients’ characteristics, symptoms severity and risk of complications [7]. Endovascular embolization is the main management technique used for DAVF with good effects, and could cause reversal of symptoms and complication risk, while curability makes this diagnosis vital to diagnosis early, even when it presents in rare but distinctive ways.

## Conclusions

Many causes of rapidly progressive dementia are reversible, especially in the early course of the disease. Bilateral thalamic venous infarction or edema should be investigated carefully for venous thrombosis. Furthermore, secondary venous thrombosis due to vascular anomalies should be suspected in patients without risk factors.
